# MicroRNA-484 is more highly expressed in serum of early breast cancer patients compared to healthy volunteers

**DOI:** 10.1186/1471-2407-14-200

**Published:** 2014-03-18

**Authors:** Silvia Zearo, Edward Kim, Ying Zhu, Jing Ting Zhao, Stan B Sidhu, Bruce G Robinson, Patsy SH Soon

**Affiliations:** 1 Cancer Genetics, Hormones and Cancer, Kolling Institute of Medical Research, University of Sydney, Sydney, Australia; 2 Hunter New England Local Health District, Royal North Shore Hospital, Sydney, Australia; 3 Department of Endocrine and Oncology Surgery, Royal North Shore Hospital, Sydney, Australia; 4 Department of Endocrinology, Royal North Shore Hospital, Sydney, Australia; 5 Department of Surgery, Bankstown Hospital and South Western Sydney Clinical School, University of New South Wales, Kensington, Australia; 6 Bankstown Hospital, Eldridge Rd, Bankstown, NSW 2200, Australia; 7 Ingham Institute for Applied Medical Research, Liverpool, Australia

**Keywords:** Breast cancer, microRNA, Serum

## Abstract

**Background:**

Previous studies have profiled breast cancer compared to normal breast tissue and identified differentially expressed microRNAs (miRNAs). These miRNAs are then assessed in serum of breast cancer patients compared to healthy volunteers. MiRNAs in serum however do not always reflect what is in tissue and important serum miRNAs may be missed. PCR arrays were therefore performed on serum samples from breast cancer patients compared to healthy volunteers with the aim of identifying circulating miRNAs that are more highly expressed in serum from early breast cancer patients compared to controls.

**Methods:**

Taqman low density array (TLDA) cards were used to profile serum miRNAs in a discovery cohort of serum from 39 early breast cancer patients compared to 10 healthy volunteers. The results were confirmed in a validation cohort of serum from 98 early breast cancer patients compared to 25 healthy volunteers using customized qPCR plates.

**Results:**

Seventeen miRNAs were found to have significantly higher levels in breast cancer serum compared to serum of healthy volunteers in the discovery cohort. Fourteen of these miRNAs were studied in the validation cohort and serum miR-484 was found to be at a significantly higher level in breast cancer serum compared to healthy volunteers.

**Conclusion:**

In this study, we found that miR-484 is significantly differentially expressed in serum of early breast cancer patients compared to healthy volunteers. We did not however find any correlation between miR-484 levels with histopathological parameters of the breast cancers. With further studies, miR-484 may prove useful as an adjunct to mammography for detection of early breast cancer.

## Background

MicroRNAs (miRNAs) are 21–25 nucleotide non-coding RNAs that negatively regulate gene expression in a sequence specific manner at a post-transcriptional level. Each miRNA controls the expression of multiple target genes, by binding to the 3’ untranslated region of target mRNAs to induce degradation of the message or inhibition of translation. Hence, miRNA expression can have a dramatic impact on cellular phenotype and function [1]. To date, there are 2578 miRNA transcripts in humans [2] with wide-ranging roles, including development, differentiation, growth and apoptosis [3].

MiRNAs have also been implicated in cancer. These miRNAs, termed ‘oncomirs’ , can act in a fashion analogous to tumor suppressor genes or oncogenes. A number of miRNAs have been found to be implicated in breast cancer [4]. MiRNA expression profile has also been reported to be associated with pathological features of breast cancer, such as tumor size, lymph node positivity, presence of vascular invasion and hormone receptor status indicating that miRNAs may play a role in defining the differences between these pathological profiles [4].

MiRNAs have been found to be present in bodily fluids [5]. They are stable in serum and appear to be protected from RNase activity. They also appear to be stable under extreme conditions which include boiling temperatures, low and high pH, multiple freeze/thaw cycles and storage for prolonged periods [6]. Serum miRNAs are therefore ideal as biomarkers.

A number of papers have reported that circulating miRNAs are able to distinguish serum from cancer patients compared to controls. Mitchell et al. demonstrated that serum levels of miR-141 were able to distinguish metastatic prostate cancer patients from healthy controls [7]. Serum levels of miR-299-5p and miR-411 have been reported to be significantly lower in breast cancer patients compared to healthy controls. For patients with metastatic breast cancer, it was noted that treatment resulted in an increase in the levels of these miRNAs approaching those of healthy controls [8].

Previous studies have profiled breast cancer compared to normal breast tissue and identified differentially expressed miRNAs. These miRNAs are then assessed in serum of breast cancer patients compared to healthy volunteers. MiRNAs in serum however do not always reflect what is in tissue and important serum miRNAs may be missed. We therefore decided to perform PCR arrays of serum samples from breast cancer patients compared to healthy volunteers. The aim of this paper was two-fold: 1) to identify circulating miRNAs that are found at higher levels in serum from early breast cancer patients compared to controls; these miRNAs may then be potentially used as serum biomarkers for identifying breast cancer patients and 2) to determine if expression of these circulating miRNAs are markers of clinical outcome such as pathological criteria and axillary lymph node status in breast cancer.

## Methods

### Patient serum samples

Ethics approval for the study was obtained from the Northern Sydney and Central Coast Area Health Service Human Research Ethics Committee, Sydney, Australia. Serum samples were obtained from the Kolling Breast Tissue Bank and Australian Breast Cancer Tissue Bank. The discovery cohort consisted of serum samples from 39 breast cancer patients and 10 healthy volunteers, while the validation cohort consisted of serum samples from 98 breast cancer patients and 25 healthy volunteers. All the breast cancer patients had operable breast cancer with no evidence of metastasis on staging investigations and who had not undergone neoadjuvant chemotherapy. For the breast cancer patients, blood was collected into a Vacuette serum clot activator gel free tube when they attended the preadmission clinic 2–4 weeks prior to the surgery or while they were in the anaesthetic bay, prior to their general anaesthetic and surgery. The blood in the tube was kept on ice for 15–30 minutes, then centrifuged for 15 minutes at 3000 rpm at 4°C. The serum was then divided into 1.5 mls cryovials in 500 μl aliquots and stored at -80°C. For healthy volunteers, blood was collected from patients at the menopause clinic into Vacuette serum clot activator gel free tubes, and processed and stored as per the protocol described above.

### RNA extraction

Small RNA was extracted from 200 μl of serum using the miRvana™ PARIS™ kit as per the manufacturer’s instructions. The quality of RNA was assessed using the Nanodrop spectrophotometer.

### Taqman low density array (TLDA) MicroRNA analysis

MiRNA profiling was performed with the TaqMan® Array Human MicroRNA Cards A and B v3.0 as per the manufacturer’s protocol, with each card quantitating 377 miRNAs. Briefly, 30 ng of RNA was initially reverse transcribed using the Megaplex RT Primers Pools A and B followed by pre-amplification with Megaplex Pre-amp Primers Pools A and B, and 800 μl of the pre-amplification product was then loaded on the TaqMan® Array Human MicroRNA Card and run on the AB7900HT. Preamplification has been shown by Mestdagh et al. to be sensitive and reliable without introducing bias [9].

Data analysis was performed using RQ Manager 1.2.1, DataAssist v3.0 and qBasePlus. MiRNAs with a C_T_ value > 37 were considered unamplified. MiRNAs in which more than 12 samples were not amplified were considered to be lowly expressed and therefore excluded, resulting in 88 miRNAs being included in the analysis. Global normalization was performed. Additional analyses were performed using miR-16 for normalization with similar results.

### Quantitative reverse transcription polymerase chain reaction (qPCR) validation

Validation of the TLDA findings was performed by qPCR using TaqMan® Custom 384-well Plates on which the following TaqMan® miRNA assays were pre-plated in triplicate: hsa-miR-16, U6 snRNA, hsa-miR-186, hsa-miR-484, hsa-miR-29a, hsa-miR-425-5p, hsa-miR-454, hsa-miR-574-3p, hsa-miR-140-3p, hsa-miR-222, hsa-let-7b, hsa-miR-483-5p, hsa-miR-21, hsa-miR-195, hsa-miR-155, hsa-miR-218. Global normalization was performed. Additional analyses were also performed using miR-16 for normalization with similar results. Thirty ng of RNA was reverse transcribed using Custom RT primer pool then pre-amplified using Custom PreAmp primer Pool and 100 μl of pre-amp product was then loaded onto the custom plates with each plate accommodating 8 samples.

### Tissue

Twelve breast cancer fresh frozen tissue and the corresponding normal breast tissue were obtained from the Kolling Breast Tissue Bank. qPCR assessment of hsa-miR-484, hsa-miR-21, hsa-miR-16 and U6 snRNA was performed on the AB7900 in triplicate.

### Statistical analysis

The microarray data was analyzed using t-test and Bonferroni correction for false discovery rate such that differential expression was considered to be significant with a p < 0.0001. The data was analyzed using QBasePlus and Data Assist. For the discovery and validation cohorts, normalization was performed using global normalization. For tissue, normalization was performed using U6, a common housekeeper used for tissue but not expressed in serum. All other statistical analyses were performed with SPSS 16 for Windows and a p < 0.05 was considered significant. Categorical data were analysed using Fisher’s exact test. The Mann–Whitney test was used for qPCR statistical analysis because the data were not normally distributed and were heteroskedastic, despite attempted data transformation.

## Results

A mean of 544 ± 499 ng of RNA was extracted from the serum samples.

### Discovery cohort

Serum samples from 39 early breast cancer patients and 10 healthy volunteers were used for miRNA expression profiling. The clinical characteristics of the breast cancer patients and the pathology of the cancers are listed in Table 1.

**Table 1:** Clinical characteristics of the healthy volunteers, breast cancer patients and pathological characteristics of breast cancers in the discovery and validation cohorts as well as tissue samples used

	**Discovery cohort**	**Discovery cohort normal**	**Validation cohort**	**Validation cohort normal**	**Tissue**
Number of patients	39	10	98	25	12
Age (mean ± SD)	58 ± 16	60 ± 6	55 ± 12	57 ± 12	59 ± 16
Subtype of cancer	39 IDC		89 IDC 9 ILC		12 IDC
Size of invasive cancer (mm)	30.5 ± 25.2		26.4 ± 14.5		43.1 ± 35.5
Lymph node negative (%)	19 (48.7%)		40 (44.9%)		5 (41.7%)
Number of positive axillary lymph nodes	3.0 ± 6.4		2.8 ± 5.4		5.6 ± 10.2
ER positive	28 (71.8%)		69 (77.5%)		8 (66.7%)
PR positive	22 (56.4%)		69 (77.5%)		6 (50.0%)
HER2 positive	6 (15.4%)		18 (20.2%)		3 (25.0%)

*Abbreviations*: IDC = invasive ductal carcinoma, ILC = invasive lobular carcinoma.

### PCR array analysis

In this study, RNA extracted from serum of both cancer patients and healthy volunteers had to be pre-amplified in order to increase the limit of miRNA detection. Preamplification of RNA samples has been done by others with good correlation of quantitative PCR results using RNA that has and has not been preamplified [9].

Comparison of cancer versus normal serum identified 17 significantly up-regulated miRNAs with corrected p-value <0.05 and foldchange >2 (Table 2).

**Table 2:** List of miRNAs significantly differentially expressed (corrected p-value < 0.05 and foldchange >2) between breast cancer and normal serum in the discovery cohort

**MiRNA**	**Foldchange**	**p-value**	**Other serum miRNA papers in breast cancer**
hsa-miR-186	6.1	<0.0001	
hsa-miR-484	5.5	<0.0001	
hsa-miR-29a	5.0	0.0001	
hsa-miR-425	4.9	<0.0001	
hsa-miR-454	4.0	<0.0001	
hsa-miR-574-3p	3.9	<0.0001	
hsa-miR-140-3p	3.2	<0.0001	
hsa-miR-222	3.1	<0.0001	
hsa-let-7b	3.0	0.0002	
hsa-miR-483-5p	2.8	0.0157	
has-miR-155	2.4	0.0078	Roth et al. [24] and Wang et al. [25] found over-expression of miR-155 in breast cancer patients
hsa-miR-126	2.3	<0.0001	Wang et al. [25] found under-expression of miR-126 in breast cancer patients
hsa-miR-146b-5p	2.3	0.0001	
hsa-miR-320	2.3	<0.0001	
hsa-miR-191	2.3	0.0002	
hsa-miR-342-3p	2.2	0.0003	
hsa-miR-486-5p	2.0	0.0003	

### Validation cohort

Our validation cohort consisted of serum from 98 early breast cancer patients and 25 healthy volunteers. The clinical characteristics of the breast cancer patients and pathology of the cancers are listed in Table 1. We performed qPCR using custom TaqMan® 384-well Plates on 14 miRNAs. We assessed the serum levels of hsa-miR-186, hsa-miR-484, hsa-miR-29a, hsa-miR-425-5p, hsa-miR-454, hsa-miR-574-3p, hsa-miR-140-3p, hsa-miR-222, hsa-let-7b and hsa-miR-483-5p because these miRNAs were significantly over-expressed in breast cancer serum compared to healthy serum in the discovery cohort. We chose to assess miR-21 because it is known to be widely over-expressed in cancer versus normal [4,10]. There is literature of increased serum levels of miR-195 in breast cancer patients [11] and miR-155 has a known role in breast cancer tumorigenesis [12]. miR-16 was used as one of the methods of normalization.

Of the 14 miRNAs assessed, we found that five of the miRNAs were significantly differentially expressed in breast cancer compared to healthy serum (Table 3). We noted that miR-16 was not differentially expressed between cancer and normal serum with mean expression level in cancer serum of 1.001 and 0.994 in normal serum. We did not find a difference in the level of serum miRNA between patients with invasive ductal carcinoma compared to the lobular subtype. We chose to concentrate on miR-484 because it was found to be significantly differentially expressed in cancer versus normal serum in the validation cohort and there was literature demonstrating that miR-484 was also differentially expressed in the serum of pancreatic cancers [13].

**Table 3:** Results of differentially expressed (foldchange and p-value) miRNAs in the validation cohort

**MiRNA**	**Foldchange**	**p-value**
hsa-miR-484	1.6	0.0026
hsa-miR-222	1.5	0.001
hsa-miR-574-3p	1.8	0.046
hsa-miR-29a	1.5	0.02
hsa-miR-195	0.6	0.0002

We also assessed expression of miR-484 in serum samples from 8 patients with ovarian cancer but did not find any difference in expression levels of these miRNAs compared to healthy controls (miR-484: RQ 1.38 p-value 0.88).

There was no correlation between serum levels of either miR-484 with tumor size, grade, estrogen receptor (ER), progesterone receptor (PR), human epidermal growth factor receptor 2 (HER2), axillary lymph node status or patient age.

### Tissue

**Figure 1:**
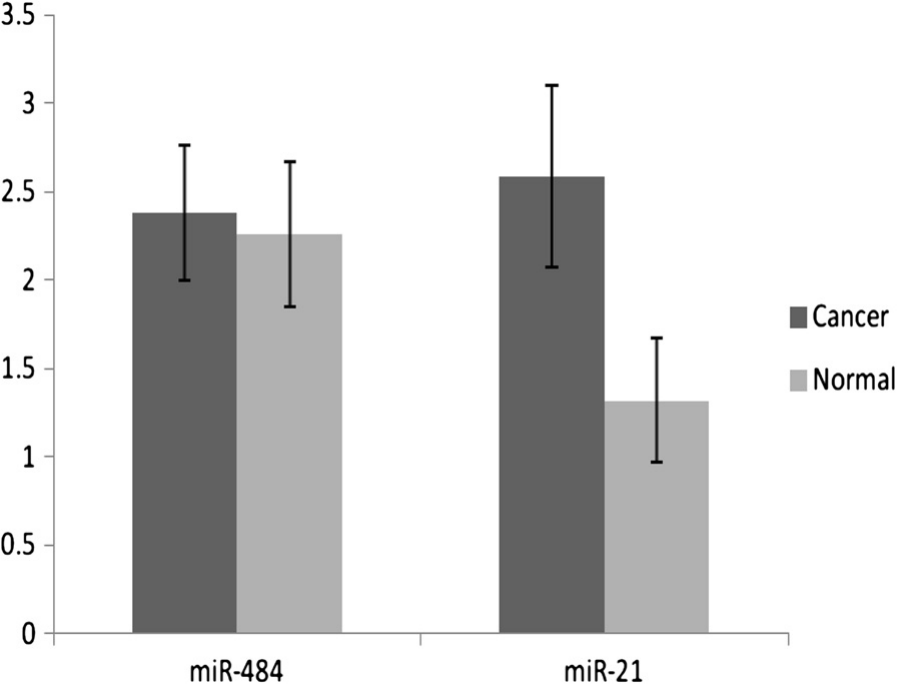
**Mean expression of miRNAs relative to U6 in breast cancer tissue (n = 12) compared to matched normal breast tissue (n = 12).** Expression of miR-21 was significantly higher in breast cancer tissue compared to normal (p = 0.02) but there was no difference in miR-484 between the 2 groups. Error bars indicate standard error of the mean.

We assessed the levels of miR-484, miR-21, miR-16 and U6 snRNA by qPCR on 12 fresh frozen breast cancer samples and the corresponding matched normal breast samples. MiR-21 has been frequently reported to be over-expressed in solid cancers [10]. MiR-16 and U6 snRNA were included as housekeepers. We found that miR-21 was significantly over-expressed in breast cancer tissue compared to matched normal tissue (foldchange 4.627, corrected p-value = 0.02), while there was no difference in expression of miR-484 between breast cancer and normal tissue samples (Figure 1).

## Discussion

The aim of this study was to identify serum miRNAs that are differentially expressed in serum of early breast cancer patients compared to healthy volunteers. We found that miR-484 was significantly differentially expressed in a discovery cohort of serum from breast cancer patients compared to healthy volunteers. These results were confirmed in a larger validation cohort.

MiR-484 has been identified to be increased in serum of pancreatic cancer patients. Li et al. compared the serum of 19 patients with pancreatic cancer compared to 10 healthy controls. They found 22 miRNAs to be significantly differentially expressed between the 2 groups. They validated 18 of these miRNAs in a different cohort of serum from 41 pancreatic cancer patients compared to 72 healthy controls. miR-484 was one of the 8 miRNAs found to be significantly differentially expressed in serum of pancreatic cancer patients compared to healthy controls [13]. This indicates that miR-484 is likely associated with a cancer process but is not specific to breast cancer. Interestingly, Li et al. found that miR-484 was more highly expressed in microdissected primary pancreatic carcinoma cells compared to normal pancreatic duct [13]. We did not however find miR-484 expression to be higher in breast cancer tissue compared to matched normal breast tissue in our small sample of 12 patients. MiR-484 expression in serum did not correlate with outcome in the pancreatic cancer patients [13].

We assessed the levels of miR-484 in serum samples from 8 patients with ovarian cancer. In contrast to the elevated miR-484 in serum samples of both breast and pancreatic cancer patients, we did not find a difference in serum levels of miR-484 in these ovarian cancer patients compared to healthy controls. These findings may indicate that miR-484 may be produced as a result of processes common to both breast and pancreatic cancer, such as desmoplasia [14,15].

Vecchione et al. found that low expression of miR-484 correlated with chemoresistance in ovarian cancer. Using *in vivo* mouse studies with miR-484 stably transduced ovarian cancer cell lines, more chemosensitive tumors resulted due to regulation of VEGFB and VEGFR2 by miR-484. The authors found that miR-484 was secreted by the ovarian cancer cells to influence the surrounding tumor-associated endothelial cells [16]. Interestingly, Volinia et al. performed survival analysis on 466 breast cancer patients by integrating mRNA, miRNA and DNA methylation data from TCGA. They found that miR-484 was one of 7 miRNAs and 30 mRNAs which formed an integrated miRNA/mRNA signature with the highest prognostic value in stage 1 and 2 breast cancers [17]. miR-484 has also been identified as one of the 10 most significantly up-regulated miRNAs in cutaneous malignant melanoma compared to benign melanocytic naevi [18].

We did not find higher levels of miR-484 in 12 samples of breast cancer tissue compared to matched normal breast tissue, which may be a result of the small sample size. In line with other reports [4,19], the expression of miR-21 was found to be significantly higher in breast cancer tissue compared to matched normal. In our study, the serum levels of miR-484 did not correlate with clinicopathological factors of the breast cancer or patient age. One study reported a correlation between serum miRNA levels and patient age but not with ER, PR or HER2 status of the cancer [8] while another study found correlation between serum miRNA levels with HER2 status but not ER or PR [20].

The origin of miR-484 in the serum in this study is unknown. Our results suggest that these miRNAs are not passively arising from the breast cancer cells. Chan et al. found that 13 miRNAs were dysregulated in breast cancer tissue and serum but in opposite direction indicating a selective release of miRNAs from the breast cancer cells or from other sources [21]. It has been suggested that miRNAs in the circulation arise from 3 possible sources – either by passive leakage from cells, such as the cancer cells or other cells including platelets or inflammatory cells; through active secretion from the cell via exosomes or microvesicles; or through active secretion from the cell via microvesicle-free, RNA-binding protein-dependent pathway [22]. Kogure et al. studied hepatocellular carcinoma cells and found that miRNAs in exosomes produced by these cells can be different from those found within the cell of origin [23].

Van Schooneveld et al. compared serum from 75 breast cancer patients and 20 healthy volunteers. They found that miR-299-5p and miR-411 were significantly under-expressed in breast cancer serum compared to serum from healthy volunteers, particularly in serum of untreated metastatic breast cancer patients [8]. Roth et al. studied 59 patients with early breast cancer, 30 with advanced breast cancer and 29 healthy volunteers. They found that expression of miR-10b, miR-34a and miR-155 was higher in breast cancer patients compared to healthy volunteers, and miR-10b and miR-34a in particular were expressed at higher levels in advanced compared to early breast cancer patients. They also did not find any correlation between the serum miRNA levels with histopathological parameters [24]. Another group found miR-21, miR-106a and miR-155 to be over-expressed and miR-126, miR-199a and miR-335 to be under-expressed in serum of breast cancer patients compared to healthy controls. This group also found correlation between tumor grade and hormone receptor status with expression of serum miRNAs [25]. Interestingly, across the different studies of serum miRNAs in breast cancer patients [8,21,24,25] including this study, there has not been a common miRNA identified which could be in part due to different RNA extraction methods and platforms used.

We used the TaqMan® Array Human MicroRNA Cards A and B v3.0 to perform miRNA analysis of our discovery cohort. Of the 384 miRNAs on card A, we only had consistent expression of 88 miRNAs. MiRNAs such as miR-10b, miR-34a and miR-199a which have previously been identified in other studies to be differentially expressed in breast cancer serum compared to healthy volunteers were not expressed in our study. There was very little expression of the miRNAs on card B. We therefore excluded card B from analysis.

Interestingly, Hu et al. performed solexa sequencing of 10 samples of pooled serum from lung, breast, cervical, gastric, hepatocellular carcinoma and healthy volunteers. They also performed qPCR using 50 other serum samples from oesophageal, colon, rectal, breast, gastric, pancreatic, oral, lung and hepatocellular carcinomas as well as healthy volunteers and found miR-484 levels to be stably expressed across all samples, leading them to recommend that miR-484 and miR-191 to be used as endogenous control for serum miRNA detection [26]. This however has not been reported by other groups.

Using DIANA-mirPath [27], a software program which identifies KEGG pathways of miRNA targets, it was found that miR-484 had 5 predicted targets which were involved in the adherens junction pathway (p-value = 0.0098). These included CDH1, the gene which encodes E-cadherin, and ERBB2, the gene which is amplified in 20-35% of breast cancer patients [28], both of which are known to be involved in breast tumorigenesis.

## Conclusion

In this study, we found that miR-484 is significantly differentially expressed between serum of early breast cancer patients compared to healthy volunteers. We did not however find any correlation between miR-484 levels with histopathological parameters of the breast cancers. With further studies, miR-484 may prove useful as an adjunct to mammography for detection of early breast cancer.

## Competing interest

The authors declare that they have no competing interests.

## Authors’ contributions

SZ and EK performed experimental work. YZ carried out bioinformatic analysis. JZ, SS and BR participated in the design of the study, provided intellectual input and critically revised the manuscript. PS conceived, designed and coordinated the study as well as drafted the manuscript. All authors read and approved the final manuscript.

## Pre-publication history

The pre-publication history for this paper can be accessed here:

http://www.biomedcentral.com/1471-2407/14/200/prepub
